# Bilateral Teleoperation of Aerial Manipulator with Hybrid Mapping Framework for Physical Interaction

**DOI:** 10.3390/s25185844

**Published:** 2025-09-19

**Authors:** Lingda Meng, Yongfeng Rong, Wusheng Chou

**Affiliations:** 1 School of Mechanical Engineering & Automation, Beihang University, Beijing 100191, China; lingda.meng@buaa.edu.cn; 2 Department of Computer Science and Technology, Tsinghua University, Beijing 100084, China; yfrong@mail.tsinghua.edu.cn

**Keywords:** bilateral teleoperation, wearable exoskeleton, unmanned aerial manipulator, hybrid mode control, physical contact task

## Abstract

Bilateral teleoperation combines the agility of robotic manipulators with the ability to perform complex contact tasks guided by human expertise, thereby fulfilling a pivotal function in environments beyond human access. However, due to the limited workspace of existing master robots necessitating frequent mapping mode switches, coupled with the pronounced heterogeneity and asymmetry between the workspaces of the master and slave systems, achieving teleoperation of the mobile manipulator remains challenging. In this study, we innovatively introduced a 7 DOFs upper limb exoskeleton as the master control device, rigorously designed to align with the motion coordination of the human arm. Regarding teleoperation mapping, a hybrid heterogeneous teleoperation control framework with a variable mapping scheme, designed for an aerial manipulator performing physical operations, is proposed. The system incorporates mode switching driven by the operator’s hand gestures, seamlessly and intuitively integrating the advantages of position control and rate control modalities to enable adaptive transitions adaptable to diverse task requirements. Comparative teleoperation experiments were conducted using a fully actuated aerial equipped with a compliant 3D end-effector performing physical aerial writing tasks. The mode-switching algorithm was effectively validated in experiments, demonstrating no instability during transitions and achieving a position tracking RMSE of 7.7% and 5.2% in the *X*,*Y*-axis, respectively. This approach holds significant potential for future applications in UAM inspection and physical operational scenarios.

## 1. Introduction

One of the primary objectives of teleoperation systems is to extend human manipulation capabilities across different scales, facilitating interaction in remote or hazardous tasks [1]. As a remote operation carrier for humans, mobile robots have seen widespread development and application across various fields with the continuous advancement of robot technology, computer science, and control theory. Within these, unmanned aerial manipulators (UAMs), typically comprising an unmanned aerial vehicle (UAV) and a robotic manipulator, enable agile three-dimensional motion [2] attributable to their remarkable maneuverability and versatility, which have received considerable attention in physical interaction tasks, including drawer operation [3], door opening [4], glass-wall inspection [5], aerial writing [6], and outdoor industrial inspection and maintenance [7]. While autonomous approaches leverage machine intelligence to perform these tasks, full autonomy is often neither feasible nor desirable due to safety concerns and the complexity of operational environments. The teleoperated UAM scheme, which employs the “human-in-the-loop” control architecture, facilitates the accomplishment of tasks in complex operational conditions under human superior cognitive capabilities and skills.

In a UAM teleoperation system, the operator employs a master robot to articulate specific intentions, including position and velocity inputs. These commands are conveyed through a communication channel to the slave UAMs, facilitating precise remote interaction with the operational environment [8]. Concurrently, force feedback and visual information perceived by the UAMs can be transported back to the human operator to assist the decision making and enhance the immersion of the teleoperation. This has garnered significant attention in research on the control of UAMs under a bilateral teleoperation scheme [9,10,11].

However, due to master–slave heterogeneity, the workspace for UAMs is not proportionally aligned with the operator inevitably. The teleoperation of UAMs predominantly employs single-mapping modes, mainly the rate control strategy, which greatly limits the application of UAMs in practical scenarios. From the literature, several studies have concentrated on hybrid teleoperation mapping schemes of ground mobile manipulators. A hybrid position–position and position–velocity control algorithm was introduced for the teleoperation of generic mobile manipulators [12]. The current progress of teleoperation mapping of mobile manipulators is summarized in [13], with consideration given to time delay and stability analysis. Such a variable mapping strategy is also extensively employed in the field of robotic arm teleoperation [14,15]. For UAV/UAM teleoperation, a variable kinetic scrolling-based mapping scheme was proposed to overcome master workspace limitations and enable teleoperation of the aerial vehicle in an unbounded workspace [16]. Additionally, a user-defined variable scale mapping controller was also presented [9]. Nevertheless, the existing UAV teleoperation mapping modes primarily focus on free movement, i.e., rate mode and position mode, neglecting contact operation modes. It is noteworthy that the transition between operational modes can potentially introduce the risk of misoperation due to complexity and potential operator errors. Therefore, developing intuitive and efficient conversion transitions between different modes based on user intention in teleoperation is crucial for advancing practical UAM applications.

Furthermore, physical contact tasks represent a significant area of focus for the future advancement of UAMs [2,17]. Implementing force feedback to perceive forces exerted by UAMs during contact tasks is recognized as an effective control approach. Shared control of path planning with integral haptic feedback was presented in [18]. Macchini et al. presented a hand-worn haptic interface to enable users to control UAV trajectories and enhance environmental awareness [19]. Meanwhile, haptic bilateral teleoperation is sophisticatedly employed in advanced UAV applications, including evaluation of obstacle avoidance [20], environmental perception [21], and nuclear sources detection [22]. Several studies have explored the bilateral teleoperation of UAMs in scenarios involving environmental interaction. Schill. et al. investigated the use of a haptic teleoperation joystick with limited workspace in admittance mode [23]. A force-based bilateral teleoperation framework was proposed both in contact-free flight and in physical contact with the environment [24]. Haptic-based shared control, incorporating artificial and virtual impedance for interaction force reflection, was designed for the telemanipulation of micro aerial vehicle (MAV) systems [25]. A team of UAMs employing a force-feedback bilateral teleoperation scheme was proposed [26], capable of cooperatively grasping objects and performing Vertical Take-Off and Landing (VTOL). A fully decoupled six DOFs bilateral teleoperation framework for omnidirectional micro aerial vehicles (OMAVs) was developed to support aerial physical interaction [10]. Byun et al. addressed the abrupt reduction in interaction force during the extraction of a wedge-shaped object (e.g., plug pulling) from a static structure, proposing a haptic-based bilateral teleoperation strategy that compensates for reaction time [11].

To improve the applicability of UAMs in physical contact tasks and ensure the stability and seamlessness of mode transitions, this paper proposes a haptic-based bilateral teleoperation scheme of a UAM for the aerial writing task. To this end, we develop an upper-limb exoskeleton as the master haptic device, configured with data glove from SenseGlove. The upper arm exoskeleton, utilized as the input device for pose control, simultaneously provides intuitive haptic feedback of the remote environment to the operator, while the application of a data glove for gesture-based switching in UAM control modes is intuitive and effective compared to traditional key-based operation [27].

Because the fully actuated UAV possesses independent control over its position and orientation, it is capable of applying force and torque in any direction with precise control over both the direction and magnitude without adjusting the fuselage posture like traditional UAVs [28]. Such UAVs are more suitable for applications such as indoor exploration in a narrow space, or as a stable platform for other air operation equipment [29]. Thus, a fully actuated UAV equipped with a 3D end-effector is utilized in this work as a teleoperation slave robot for aerial writing tasks. Based on the actual task situation, the aerial writing task was divided into free movement and contact working phases.

When in the free movement phase, the UAM is controlled in the rate mode and the pose of the master is mapped to a possibly scaled velocity reference of the slave. In this mode, UAM possesses a theoretically infinite operational space, enabling approach of the target position expeditiously and effectively. Subsequently, during the contact phase, haptic feedback is proposed as cues to indicate the teleoperated state of the slave. Also, a transition algorithm between the above two mapping methods for the master haptic device is developed to achieve smoothing switches. As a case study for the aerial writing task, two sets of experiments were conducted with the teleoperation structure. The experimental results demonstrate that the teleoperation method incorporating mapping switching enables rapid execution of contact operations. Overall, the key contributions are as follows:

(1) The development of a novel UAM teleoperation framework utilizing an exoskeleton as the master control robot.
(2) Proposing a mode-switching teleoperation mapping algorithm, with mode transitions facilitated by gestures captured through data gloves.
(3) Experimental validations on the aerial writing teleoperation experiment based on the theoretical framework.

The rest of the article is organized as follows: The system structure is presented and defined in Section 2. Section 3 provides a comprehensive examination of the two mapping modes and transition algorithms, in addition to a discussion on the passivity of the control method. Section 4 presents the validation of simulation and experimental results, demonstrating the efficacy and performance of the proposed UAM teleoperation structure. Finally, Section 5 concludes this article.

## 2. Teleoperation System Overview

**Figure 1:**
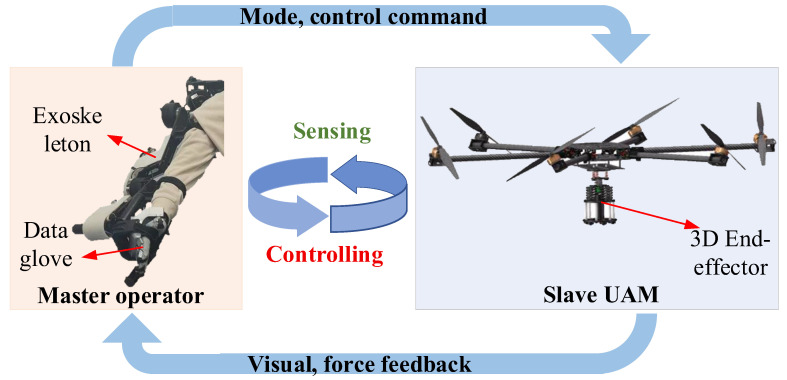
Schematic diagram of teleoperation system.

We focus on the representative scenario illustrated in Figure 1, in which a 7 DOFs upper limb exoskeleton and the data gloves are employed as master devices to teleoperate a UAM equipped with a 3D end-effector to accomplish aerial writing tasks. Visual and *Z*-axis contact data from the UAM are transmitted to the master operator via the communication channel, meanwhile enabling the operator to execute mode-switching and control commands to the UAM.

### 2.1. Exoskeleton Master Robot

In this section, the master endoskeleton detailing the structural design and control framework are introduced. Additionally, forward kinematics analysis and a description of the Bluetooth glove employed for hand data acquisition are presented.

#### 2.1.1. Functional Characterization of the Upper-Limb Exoskeleton

**Figure 2:**
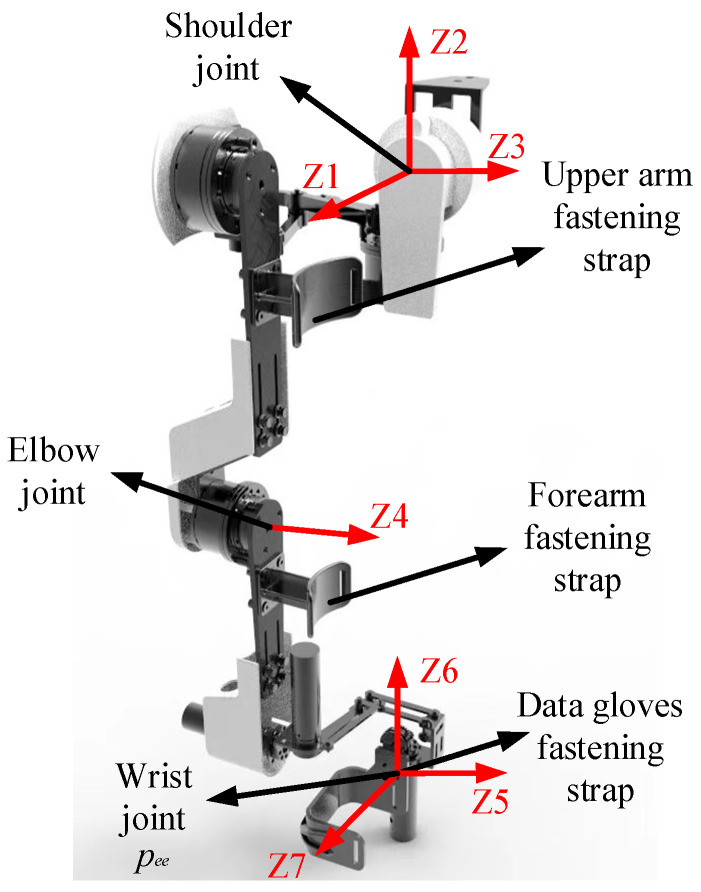
Upper arm exoskeleton robot.

In a bilateral teleoperation robot system, the master device is required to simultaneously capture real-time data on the motion of the operator’s upper limb and deliver force-feedback signals to the operator. Current commercial master devices can be primarily categorized into series input equipment, such as Phantom from Geomagic Touch [30], and parallel input equipment, such as Sigma from Force Dimension [31]. Nevertheless, the limited compatibility with human anatomy necessitates extensive training to achieve a satisfactory level of performance. Furthermore, the master robots mentioned above are unsuitable for large-scale workspaces due to their lack of intuitive interaction with the human arm. Therefore, in this study, an ergonomic upper limb exoskeleton is developed as the master input device, as shown in Figure 2.

The upper limb exoskeleton features 7 active DOF configurations to support shoulder, elbow, and wrist as SRS (spherical-rotation-spherical) joints. Each joint is equipped with encoders that measure the movements of the human arm, enabling the calculation of the end-effector *p_ee_* position. The exoskeleton is attached to the human upper limb at three key locations: the upper arm, the lower arm, and the mounting bracket for the Bluetooth data gloves. The innovation of this exoskeleton mechanical arm lies in the replacement of conventional capstan gears in the shoulder and wrist joint mechanisms with a double parallelogram spherical mechanism (DPM), which ensures precise alignment of the torque axis with the human operator axis while significantly reducing the overall weight of the exoskeleton arm. Additionally, active motors are fitted for each joint and counteract gravitational forces and provide feedback force in all three Cartesian directions (XYZ) as well as about roll, pitch, and yaw (RPY) orientations. Our exoskeleton delivers the highest continuous torque-output-to-weight ratio compared to state-of-the-art teleoperation exoskeletons.

To facilitate high-speed control of the master exoskeleton, the system employs an EtherCAT bus and achieves a control rate update of 1 kHz. The Beckhoff CX5140 Embedded PC integrating TwinCAT 3 (from Beckhoff, Wil City, Germany) establishes communication with motor drivers Elmo G-POLTWI10/100 (from Elmo, Tel Aviv, Israel). The local computer model ARK-3530F (from Advantech, Kunshan, China) interacts with the Beckhoff PC via the ADS server. The high-level control algorithms including kinematic calculation, mode mapping and force feedback mapping algorithm are executed on the local computer and communicate with the motor drivers through the TwinCAT3 (from Beckhoff, Wil City, Germany) ADS router API. This configuration enables real-time execution of advanced control algorithms and direct management of the underlying driver.

#### 2.1.2. Kinematic Analysis of the Upper Exoskeleton

The exoskeleton incorporates seven active degrees of freedom (DOFs), with the shoulder, elbow, and wrist joints configured as a serial spherical-rotational-spherical (SRS) kinematic chain. The sternoclavicular joint on the right side is selected as the base, with *l_a_* = 42 mm and *l_b_* = 175 mm representing the distances from sternoclavicular joint to the glenoid joint in the sagittal and coronal planes, respectively. The lengths of the upper arm *l_c_* = 265 mm, forearm *l_d_* = 315 mm, and palm length can be adjusted by the passive prismatic joints to accommodate variations in the operator’s height. The system is engineered to accommodate operators with statues ranging from 165 cm to 185 cm, ensuring optimal ergonomic compatibility. The kinematic model of the exoskeleton is developed using modified Denavit–Hartenberg parameters, as presented in Table 1. The right column of the table specifies the allowable range of joint rotation angles for the robotic exoskeleton.

**Figure 3:**
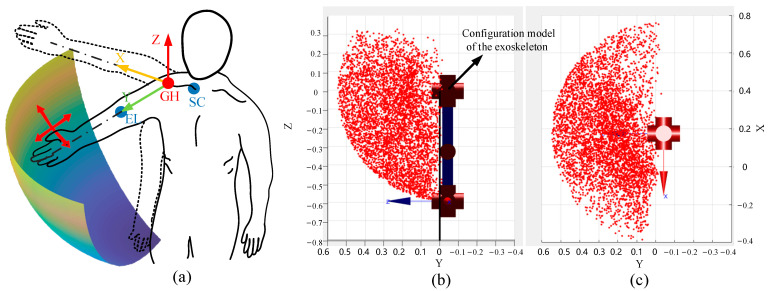
Upper arm exoskeleton workspace. (**a**) Reachable workspace. (**b**) Illustration of workspace using Monte Carlo in sagittal plane. (**c**) Workspace in horizontal plane.

The Monte Carlo method is utilized to calculate the effective workspace of the master exoskeleton. Joint angles are incorporated as variables within the forward kinematics model, and the workspace is discretized into a series of cells through slicing along the *X*, *Y*, and *Z* axes. As depicted in Figure 3b, the red 3D point cloud represents the working space accessible by the center point of the right wrist joint. The working space of the exoskeleton approximates a hemispherical volume, aligning with the operational space anterior to the human body. The diameter of this hemispherical workspace varies with the length of the operator’s arm, achieving a maximum radius oof 629 mm, as depicted in Figure 3a. Notably, to address the practical requirements of teleoperation, only the motion space corresponding to the anterior coronal plane is extracted. In contrast to heterogeneous robotic arms, the UAM is theoretically capable of possessing an infinite workspace; therefore, traditional workspace mapping methods as described in [30] are insufficient. Detailed control strategies will be comprehensively addressed in the following section.

#### 2.1.3. Data Gloves

**Figure 4:**
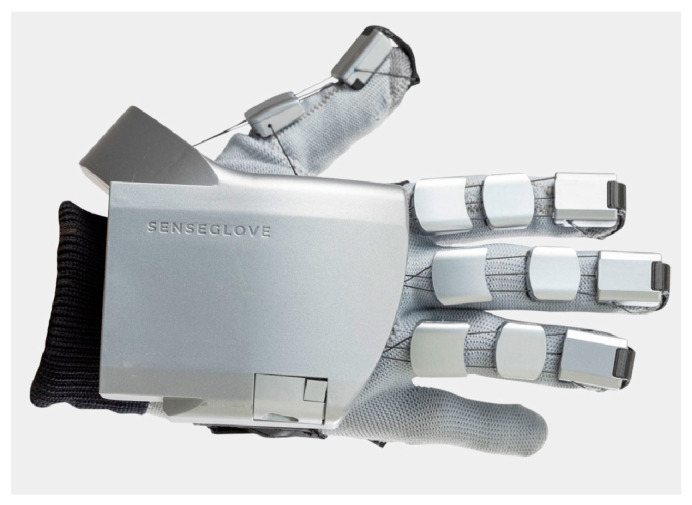
Data glove from SenseGlove Nova.

Prior research on mode switching during teleoperation has primarily utilized button-based interfaces, which can lead to operator disorientation regarding the current operational mode, potentially compromising system stability. This study proposes addressing this issue by employing data gloves to track the operator’s hand movements and assign distinct hand gestures to specific modes. This approach not only enhances the range of motion mapping modes but also provides an intuitive human–machine interface. To achieve this, the investigation utilizes the SenseGlove Nova Bluetooth data gloves, which measure the flexion and extension of the thumb, index, middle, and ring fingers using individual sensors, with an additional sensor for thumb abduction and adduction, as illustrated in Figure 4. The pinky finger’s flexion is mechanically coupled to that of the ring finger. These movements are captured by monitoring the extension of cables integrated into the gloves.

The data glove interfaces with the local computer via Bluetooth 4.2 protocol and samples cable extensions at a rate of 60 Hz with a resolution of 0.03 mm. The latency for detecting finger movement and updating the position ranges from 10 to 29 ms, while the latency for issuing a new command and its activation on the glove ranges from 15 to 22.5 ms. To accommodate the practical requirements of this study, latency issues are not considered, and thus, delays arising from data acquisition and transmission are neglected.

### 2.2. Teleoperation Slave Robot

In this section, a comprehensive description of the slave robot designed for aerial writing is provided, with the structural control framework of the fully actuated UAM and the 3D end-effector to accommodate the offsets in all rotational axes.

#### 2.2.1. Fully Actuated UAV

**Figure 5:**
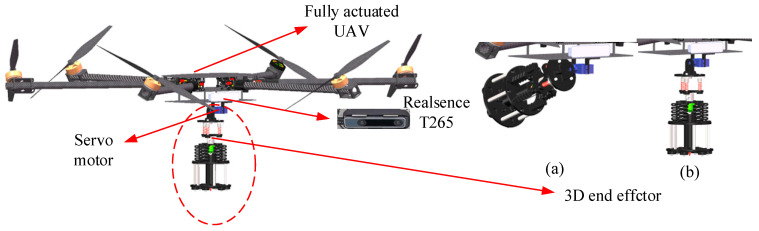
Slave UAM structure diagram. (**a**) The folded state of the 3D end-effector; (**b**) the extended state of the 3D end-effector.

The fully actuated UAV is transformed from a traditional hexarotor by tilting each propeller 30 degrees about the mounting arm [32]. The UAV features a diagonal wheelbase of 1000 mm, with each propeller capable of generating a maximum thrust of 15 N. The actuator dynamics of the motor is tested using a 1D force sensor capable of measuring force along the *Z*-axis, following a methodology akin to that in [33], and the average time constant is α = 0.08. The open-source Pixhawk flight controller generates PWM commands for the UAV motors and manipulator servos, while odometry feedback is provided by a RealSense T265 camera (from Intel, Santa Clara, CA, USA). The control algorithm operates at a frequency of 100 Hz on an onboard computer model 11TNHi7 from Intel interfaced with the Pixhawk2.4.8 via USB. A 24 V tethered power supply sustains the operation of the system. The inertia of the UAV is set constantly to *J*_u_^B^ = diag {0.2, 0.45, 0.45} (kg·m^2^). The structure of the remotely controlled UAM is depicted in Figure 5. The 3D end-effector is mounted on the underside of the UAM’s fuselage and is controlled by a servo motor to transition between the folded state and the extended state during takeoff and landing phases. The design of the 3D end-effector is elaborated in detail in the subsequent section.

#### 2.2.2. Design of the 3D End-Effector

A 3D end-effector is affixed to the UAV and internally equipped with a whiteboard pen for aerial writing. To accommodate the offsets in all rotational axes and ensure that the whiteboard pen is physically perpendicular to the whiteboard, a spherical joint is implemented, as well featuring a universal ball beneath the contact plate, which facilitates multidirectional rolling.

**Figure 6:**
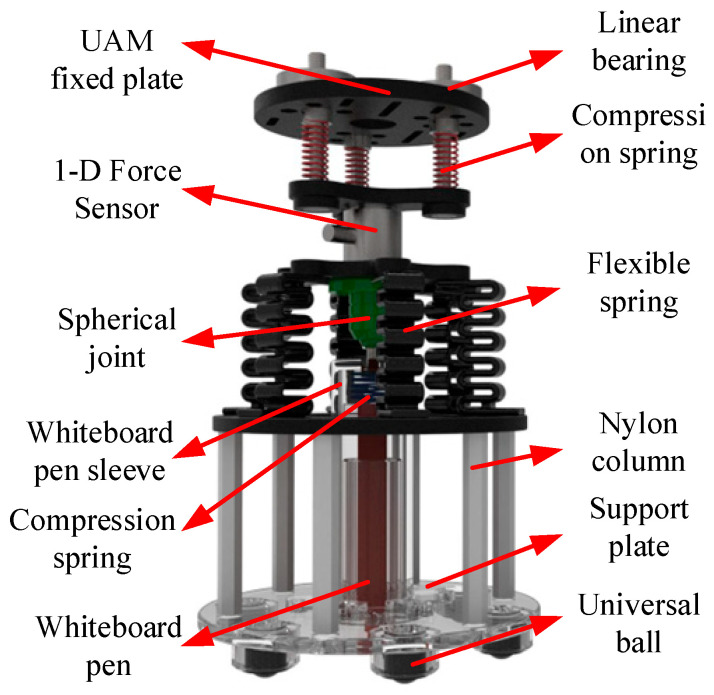
Three-dimensional end-effector structure diagram.

As illustrated in Figure 6, the 3D end-effector structure design can be segmented into three layers from top to bottom. The top layer comprises three linear bearings and springs in a circular arrangement, acting as a damper to isolate *Z*-axis disturbances. A 1-D force sensor with a measurement range of 0–20 N and with an accuracy of 0.1 N is mounted below this to measure the end-effector interaction force alone UAM’s Z direction. The middle layer features a ball joint connecting the 1-D force sensor to the marker holder, along with six flexure springs to stabilize rotational movements and maintain the marker’s perpendicular alignment with the writing surface. The bottom layer houses the whiteboard marker within a sleeve, with a compression spring ensuring consistent surface contact. Universal balls located beneath the support disk allow the end-effector to glide freely on the tablet. To optimize the maneuverability of the UAM, the integrated module, with a minimal mass of only 580 g, is securely mounted to the ventral surface of the unmanned aerial vehicle. The system is driven by a servomotor to reorient to a vertical contact plane under stabilized operational conditions.

### 2.3. Teleoperation Scheme

**Figure 7:**
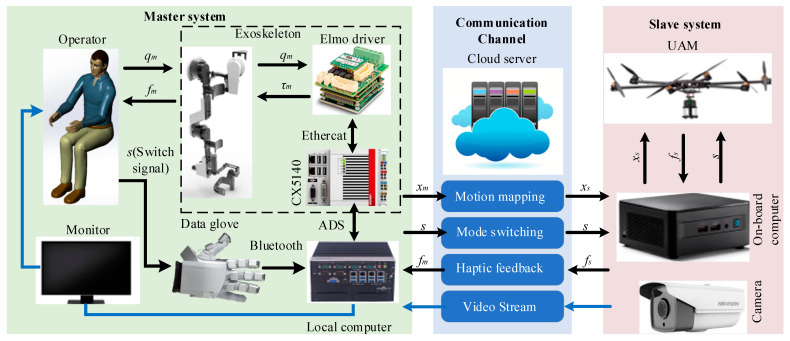
Teleoperation structure diagram.

The architectural framework of the exoskeleton–UAM teleoperation system is illustrated in Figure 7, comprising a master system, a slave system, and a communication channel. The master system incorporates an exoskeleton engineered to capture the operator’s motion data with precision and deliver haptic feedback, effectively transmitting interaction forces from the remote environment to the operator. In addition, the master system also integrates the human operator, a monitor for displaying returned image information, data gloves for capturing the operator’s hand movements, a local computer that facilitates communication between the data gloves and the Beckhoff CX5140 embedded PC, and the master device. The slave system includes the fully actuated UAM with the 3D end-effector and the remote environment. The slave system is equipped with a camera for real-time visual data acquisition, enabling the continuous monitoring of dynamic changes in both the slave-end equipment and its surroundings. A data exchange stream uses a separate data channel from the video channel, with tests confirming a signal latency of less than 10 ms; consequently, teleoperation system delays are not addressed in this study.

The local computer in the master system incorporates control modules for kinematic analysis, spatial mapping algorithms, haptic feedback control, and mode-switching capabilities. The terminal position of the wrist joint center point is calculated through the angular motion of the operator’s upper limb and mapped to the UAM’s motion using a spatial mapping algorithm. To mitigate instability due to operator input jitter, mean filtering is applied to the positional signals derived from kinematic calculations. Haptic feedback control is then employed to complete aerial rigid contact operation. In the slave system, a novel adaptive extended-state-observer-based (AESO-based) impedance control method, as introduced in [34], is implemented to regulate the UAM position.

## 3. Model and Method

To enhance the remote operability of UAMs, intuitive and accessible human–robot mapping strategies are critical for achieving superior maneuverability. Harnessing human intelligence for mapping transformations enables effective control within the constrained workspace of the master robot. A dynamic mapping approach is proposed, integrating free-rate and position-based modes, effectively addressing the challenges of mapping between constrained and unconstrained spaces. Furthermore, the incorporation of force feedback is under consideration to augment operational flexibility, precision, and efficiency in complex task execution.

### 3.1. Free Rate Mode

Rate control has proven suitable for UAMs during unconstrained motion. Within the remote manipulation framework of this study, the free rate mode of UAM operation is employed at the prepare position, typically remote from the operational area, to enable swift transit to the designated target location. However, when the slave UAM is operated in the rate control mode, significant stability and transparency issues are experienced upon contacting the environment [35]. Notably, due to the serial configuration of the exoskeleton master robot, changes in the position of the end-effector also influence its orientation and the attitude adjustment range of the UAM is limited during operations. Consequently, we employ a decoupled mapping strategy for teleoperation: the UAM positional movements are mapped through the shoulder and elbow joint position illustrated in Figure 2, while the orientation is directly controlled by three wrist joints. This decoupled attitude–position mapping approach also effectively mitigates singularities in robotic control systems and ensures that the UAM orientation will not change along with the variation in the end position. This decoupling may increase control complexity and limit the system’s overall flexibility. Nevertheless, these trade-offs are effectively counterbalanced by the significantly enhanced precision of UAM control and the expanded effective operational range achieved through mode switching.

**Figure 8:**
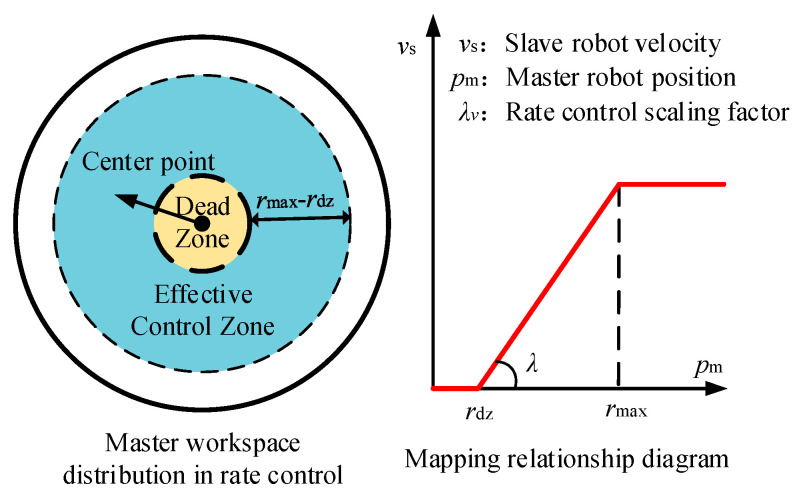
Master pose and mapping velocity in free rate mode.

To avoid misoperation by the operator’s input, a dead zone is defined around the center of wrist joint *p_ee_*, as illustrated in Figure 8. The velocity of the UAM is characterized as a vector with a magnitude directed outward from the central point. A maximum operational region *r*_max_ is defined, and when the input from the master robot surpasses the boundary, the slave robot is instructed to maximum velocity. In the free rate mode, the position and wrist joint angle denoted as $H_{ee}^{0}$ is mapped to a scaled twist reference for the slave manipulator as follows:

(1) $$T_{\mathrm{rate}}^{0}=\Phi_{\mathrm{rate}}(H_{ee}^{0})=\left[\begin{array}{c} \mathrm{diag}(\lambda_{v\theta })(\theta_{ee}^{0}(t)-\theta_{dz}^{n}-\left\|\theta_{ee}^{0}(t)-\theta_{dz}^{n}\right\|r_{v\theta })+\alpha_{n-1,v\theta }\\ \mathrm{diag}\left(\lambda_{vt}\right)\left(p_{ee}^{0}(t)-p_{dz}^{n}-\left\|p_{ee}^{0}(t)-p_{dz}^{n}\right\|r_{vt}\right)+\alpha_{n-1,vt} \end{array}\right]$$

with

(2) $$\theta_{dz}^{n}=\theta_{ee,t_{2}}^{0},\;p_{dz}^{n}=p_{ee,t_{2}}^{0}$$

where *λ* = [*λ_vθx_*, *λ_vθy_*, *λ_vθz_*, *λ_vx_*, *λ_vy_*, *λ_vz_*]^T^ is the scaling factor of angle and position of end-effector. In the free rate mode, position commands are transmitted to control velocity via scaling factors *λ_v_*, as illustrated in Figure 8; whereas in the position mode, the scaling factors *λ_p_* serve as workspace mapping adjustment parameters. *α*_*n*−1_ = [*α_θx_*, *α_θy_*, *α_θz_*, *α_x_*, *α_y_*, *α_z_*]^T^ is a vector representing the desired pose offset between the center point of the master end-effector and UAM, respectively. *θ_ee_* and *p_ee_* denote the wrist joint angles and central position of the end-effector. Similarly, *θ_dz_* and *p_dz_* denote the sphere center of position and rotation and *r_θ_* and *r_t_* denote the coordinate range of the designated dead zone.

To enhance the efficiency and intuitiveness of UAM teleoperation, we integrate reset and normal modes within the mapping framework. Under optimal conditions in the normal mode, the operator initiates teleoperation from a neutral pose, where the effective control zone resides within the master robot’s workspace, and effective control of the UAM becomes infeasible. Therefore, inspired by the computer mouse, the cursor remains stationary when the mouse is lifted from the surface, regardless of its position. Meanwhile, contact with the surface sets a new zero-point for control. The reset mode in this study suspends teleoperation to recalibrate the neutral center of the mapping between the master and slave workspaces, ensuring precise alignment and effective control. In reset mode, the master robot’s neutral pose is redefined to align with the UAM’s current position, recentering the effective control zone within the master’s workspace while the UAM maintains its position and orientation. Once recalibration is complete, the operator resumes control in normal mode with an adjusted workspace, enabling seamless and intuitive operation in challenging environments.

In this research, we establish two mapping submodes by altering the offset α of the pose of the master when instruction is issued at n-th switching time. In the reset mode, the desired offset α represents the pose at the time of switching (*t_n_*), as shown in Equation (4). When switching from position mode to reset mode at *t_n_*, the system records the instantaneous pose data. While in reset mode, the dead zone center dynamically tracks the motion trajectory, whereas UAM control commands remain constant. Upon transitioning back to position mode, the system redefines the dead zone center by leveraging the positional data recorded at the time of mode transition *t_n_*_+1_, thereby effectively repositioning the center of the dead zone to match the normal mode perceived pose. The alpha values for the two submodes are denoted as follows:

(3) $$\alpha^{n}=\left\{\begin{array}{c} 0\;normal\\ -\left[\begin{array}{c} diag(\lambda_{v\theta })(\theta_{ee}^{0}(t)-\theta_{dz}^{n}-\left\|\theta_{ee}^{0}(t)-\theta_{dz}^{n}\right\|r_{v\theta })\\ diag\left(\lambda_{vt}\right)\left(p_{ee}^{0}(t)-p_{dz}^{n}-\left\|p_{ee}^{0}(t)-p_{dz}^{n}\right\|r_{vt}\right) \end{array}\right]reset \end{array}\right.$$

with

(4) $$\theta_{dz}^{n}=\theta_{dz,t_{n}}^{n},\;p_{dz}^{n}=p_{dz,t_{n}}^{n}.$$

### 3.2. Pose Mode

Rate mapping enables teleoperation across unbounded spaces but lacks the precision necessary for operational tasks. In contrast, pose mapping provides enhanced precision for contact tasks within a constrained workspace. In the pose mode, the end-effector’s pose of the master robot is mapped to the central pose of the UAM through a scaling factor. Specify the homogeneous matrix representations for the orientation of the master exoskeleton’s end-effector, with the mapping function defined as follows:

(5) $$H_{pose}^{0}=\Phi_{pose}(H_{ee}^{0})=\left[\begin{array}{cc} R_{ms}^{0}(t) & p_{ms}^{0}(t)\\ 0 & 1 \end{array}\right].$$

with

(6) $$\begin{array}{l} R_{ms}^{0}(t)=\left[\begin{array}{ccc} 1 & 0 & 0\\ c\left(\theta_{t_{x}}\right) & -s\left(\theta_{t_{x}}\right) & 0\\ 0 & s\left(\theta_{t_{x}}\right) & c\left(\theta_{t_{x}}\right) \end{array}\right]\left[\begin{array}{ccc} c\left(\theta_{t_{y}}\right) & 0 & s\left(\theta_{t_{y}}\right)\\ 0 & 1 & 0\\ -s\left(\theta_{t_{y}}\right) & 0 & c\left(\theta_{t_{y}}\right) \end{array}\right]\\ \;\left[\begin{array}{ccc} c\left(\theta_{t_{z}}\right) & -s\left(\theta_{t_{z}}\right) & 0\\ s\left(\theta_{t_{z}}\right) & c\left(\theta_{t_{z}}\right) & 0\\ 0 & 0 & 1 \end{array}\right]\\ p_{ms}^{0}(t)={\left[diag(\lambda_{pt})(p_{ee}^{0}(t)-p_{dz}^{n}-\left\|p_{ee}^{0}(t)-p_{dz}^{n}\right\|r_{t})+\alpha_{n-1,pt}\right]}^{T},p_{dz}^{n}=p_{dz,t_{n}}^{n} \end{array}$$

where *c*(.) and *s*(.) represent the cosine and sine functions. **R***_ms_*^0^(*t*) and *p_ms_*^0^(*t*) denote the rotation matrix and position vector relative to the *X*, *Y*, *Z* axes. Noting that, the rotation matrix adheres to the ZYX Euler angle convention, where rotations are performed sequentially about the *Z*-axis (yaw, *ψ*), *Y*-axis (pitch, *θ*), and *X*-axis (roll, *φ*) with respect to the master wrist joint 7, 6, 5. A dead zone is also implemented in the pose mode to minimize vibrations during teleoperation both for position and orientation. The mapped waist angle, denoted as ***θ****_t_*, aligns with the angular representation provided in Equation (1).

(7) $$diag(\lambda_{p\theta })(\theta_{ee}^{0}(t)-\theta_{dz}^{n}-\left\|\theta_{ee}^{0}(t)-\theta_{dz}^{n}\right\|r_{p\theta })+\alpha_{n-1,p\theta }$$

with

(8) $$\theta_{dz}^{n}=\theta_{dz,t_{n}}^{n}$$

In pose mode, a larger scaling factor *λ_p_* can obtain a greater position range but also diminish the control accuracy. A significant challenge in the other research lies in achieving an optimal balance between control accuracy and spatial extent. A dual-submode switching approach, analogous to speed mapping, is employed to enhance precision. In normal mode, the effective control zone is directly confined to the master workspace. The discrepancy between the master and the dead zone, as specified by the operator, represents the desired offset relative to the zero pose of slave UAM.

Similarly to the reset mode in free rate mode, through the pose reset mode, the origin of the master and slave workspaces is recalibrated. This recalibration aligns the slave’s posture, as perceived by the operator during mode switching, with a neutral posture that serves as the desired reinitialized reference between the master and slave workspace. Effectively, this process recenters the achievable workspace of the slave to this posture when the operator perceives it. In this state, the teleoperation workspaces of the master and slave are asymmetric. We designate this sub-mode as the pose reset submode, where the offset α in the two sub-modes of the posture mode can be expressed as follows:

(9) $$\alpha^{n}=\left\{\begin{array}{c} 0\;normal\\ \left[\begin{array}{c} \theta_{dz}^{n}-diag(\lambda_{p\theta })(\theta_{ee}^{0}(t)-\theta_{dz}^{n}-\left\|\theta_{ee}^{0}(t)-\theta_{dz}^{n}\right\|r_{p\theta })\\ p_{dz}^{n}-diag\left(\lambda_{pt}\right)\left(p_{ee}^{0}(t)-p_{dz}^{n}-\left\|p_{ee}^{0}(t)-p_{dz}^{n}\right\|r_{pt}\right) \end{array}\right]reset \end{array}\right.$$

with

(10) $$\theta_{dz}^{n}=\theta_{dz,t_{n}}^{n},\;p_{dz}^{n}=p_{dz,t_{n}}^{n}$$

### 3.3. Mode Transformation

**Figure 9:**
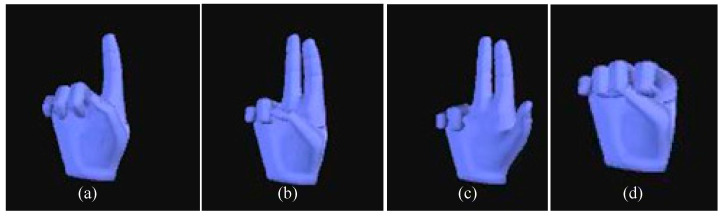
Mode transformation by hand gesture recognition. (**a**) Free rate normal mode; (**b**) pose mode; (**c**) force feedback mode; (**d**) reset mode.

In this section, the transition algorithm for switching between the two workspace mapping methods is developed and described in detail. The Novasense Bluetooth data glove is utilized as the mode switching device capable of sensing and receiving hand command information from the operator intuitively. Gesture one depicted in Figure 9a serves as the control signal for the rate mode, and gesture two shown in Figure 9b acts as the switching signal for the position mode. The gesture illustrated in Figure 9d represents the reset mode, while the gesture depicted in Figure 9c denotes force feedback mode which is described in detail in the next section. The switch controlled by the operator can be performed when mode transformation is necessary. Compared to conventional button-based switching modes, the data glove enhances the flexibility of the mapping algorithm and offers diverse switching options. By enabling direct operator control over mode switching, the teleoperation mapping achieves greater transparency.

To prevent conflicts between the speed mode and position control mode, a binary logical input *s* is introduced to denote the operational mode, where *s* = 0 corresponds to the position control mode and *s* = 1 corresponds to the speed mode. To ensure the safety of the teleoperation process, transitions between the speed control mode and the rate control mode are restricted, requiring passage through the reset mode. Conversely, the pose control mode and the force feedback mode may be interchanged freely during operation. Upon switching to force feedback mode, it is postulated that the UAM is in contact with the whiteboard surface, thereby fixing the *Z*-axis position at the height established during the mode switch, with no teleoperation control applied to the *Z*-axis. Furthermore, force feedback mode incorporates haptic feedback, while the *X*,*Y*-axis teleoperation mapping algorithm remains consistent with that employed in position mode. The mapping function is expressed by the following equation:

(11) $$\Phi \left(s,H_{ee}^{0}\right)=(1-s)\Phi_{\mathrm{pose}\;}\left(H_{ee}^{0}\right)+s\Phi_{\mathrm{rate}\;}\left(H_{ee}^{0}\right)$$

The proposed mapping strategy allows for continuous and seamless switching between two sub-modes. Switching transformation signals are sent under the operator’s control whenever necessary, enhancing the flexibility of the mapping algorithm. In contrast to button-based switching, the data glove utilized in this research offers an intuitive approach and minimizes the probability of operational errors due to mode switching.

### 3.4. Force Feedback in Physical Contact Tasks

The present research proposes a haptic feedback framework aimed at enhancing performance and operator immersion in human–robot interaction systems. Within the established position-mode teleoperation framework, pose commands from the master are directly mapped and transmitted to the contact end of the unmanned autonomous manipulator (UAM).

**Figure 10:**
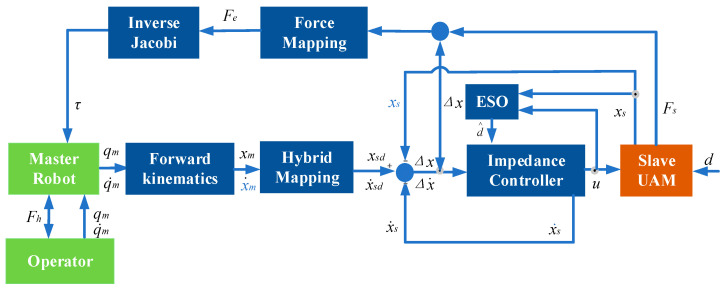
Force feedback control based on impedance control.

When the end-effector of the slave UAM encounters a rigid object, minor positional errors may generate substantial contact forces, potentially undermining the stability and safety of the teleoperation system. To mitigate this, a force feedback framework based on impedance control, as depicted in Figure 10, is introduced.

Through the master–slave pose mapping algorithm, the position commands of the master device *x_m_* are transformed into the desired position of the slave device *x_s_*. The characteristics of a spring-damper second-order system are integrated into the impedance model of UAM, and the impedance controller is formulated as follows:

(12) $$\begin{array}{l} M_{d}\Delta {\overset{..}{x}}_{s}+D_{d}\Delta {\dot{x}}_{s}+K_{d}\Delta x_{s}=F_{s}\\ \Delta x=x_{sd}-x_{s} \end{array},$$

The dynamics model of the slave UAM can be represented as follows:

(13) $$\begin{array}{l} u_{d}-u_{s}=u_{d}-T_{fs}F_{s}\\ =M_{s}(q)\overset{..}{q}+C_{s}(q,\dot{q})\dot{q}+G_{s}(q) \end{array}$$

where *F_s_* indicates the detect force from the force sensor. **K***_d_* is the stiffness parameter; **D***_d_* is the damping parameter; **M***_d_* is the inertia parameter; **M***_s_* is the inertia matrix. $C_{s}(q,\dot{q})$ is the Kohl force matrix; **G***_s_*(*q*) is the gravity matrix of UAM and **T***_fs_* represents the conversion matrix that transforms the external force into a control value. The term u*_d_* represents the control input for the UAM generated by the control framework to regulate the UAM’s dynamic. Conversely, u*_s_* denotes the external control value transmitted to the UAM, which accounts for external forces or disturbance. In addition, the UAV is subject to external disturbances *d*, such as wind, during task execution. To address this issue, an extended state observer (ESO) is incorporated into the control system of the UAM to enhance stability [36]. Finally, the following impedance control calculation can be obtained as follows:

(14) $$\begin{array}{l} \tau_{d}=J_{s}^{T}\left(M_{d}\Delta \overset{..}{x}+D_{d}\Delta \dot{x}+K_{d}\Delta x\right)\\ +M_{s}(q)\overset{..}{q}+C_{s}(q,\dot{q})\dot{q}+G_{s}(q) \end{array}$$

Feedback force *F_f_* comprising the contact force *F_s_* from the slave environment and the difference *p_e_* between the desired and actual positions of the UAM are transmitted to the master robot through the force mapping channel as follows:

(15) $$F_{f}{=}_{s}^{m}K_{f}\left(K_{e}p_{e}+K_{s}F_{s}\right)$$

where *K_s_* and *K_e_* denote the coefficient of the contact force and impedance force because of position error. Meanwhile, *K_f_* denotes the force feedback mapping coefficient, which scales the combined impedance forces and contact force to the force feedback output. In the experiment, *K_s_* and *K_f_* were set as 1 to ensure the transparency of force feedback, while *K_e_* was set as 50 N/m. The teleoperation framework presented in this paper is decoupled for position and orientation. Specifically, the first four joints of the master exoskeleton are responsible for position control and feedback three-dimensional contact forces, while the three motors in the wrist joint control the orientation. The end-effector is equipped with a 1-D force sensor, which detects the contact force in the vertical direction between the UAM and the operating environment. The relationship between the feedback force *F_f_* and the exoskeleton motor torque ***τ***_m_ can be represented as follows:

(16) $$\tau_{m}={J_{m}}^{T}\cdot F_{f}$$

where **J**_m_ denotes the Jacobian matrix of the exoskeleton. The inverse kinematics solution for the 7-DOF robotic system involves redundancy, resulting in multiple feasible configurations. To address this, the present study incorporates an additional constraint, as defined in Equation (17), which minimizes joint torque output to identify the optimal solution. The term ***τ****_i_* specifically represents the force feedback applied to the shoulder and elbow joints of the exoskeleton, excluding the total output torque, as it also accounts for gravity compensation within the exoskeleton.

(17) $$\mathrm{Minimize}\begin{array}{c} Q=\sum_{i=0}^{n}{\left\|\tau_{i}\right\|}^{2} \end{array}$$

The teleoperation system typically consists of the operator, the master robot and controller, the communication channel, the slave robot and controller, and the operating environment. Accordingly, establishing the theoretical stability of the teleoperation system is complicated and challenging. Alternatively, passivity theory presents an approach to establish system stability from the energy perspective [37]. The total energy is dissipated but not increased in a passive system [38]. In the passive system, the stored energy remains bounded by the sum of the initial energy storage and the energy supplied to the system which is expressed as follows [39]:

(18) $$V(t)-V(0)\leq \int_{0}^{t}W(t)dt,\forall t>0$$

where *W*(t) represents the real-valued rate of energy, corresponding to the input power supplied to the system. *V*(0) is the initial energy, while *V*(t) is the stored energy function. The stability of the interconnected systems can be rigorously established through passivity theory.

In the teleoperation system, the operator refrains from deliberately compromising system stability and adopts a passive response to external inputs. Concurrently, both the human operator and the environment can be modeled as passive systems [40]. Consequently, both the master and slave robots demonstrate passivity with respect to the human operator and the environment. Additionally, a cloud server is incorporated into the master–slave system to facilitate data transmission. The data stream is transmitted independently of the video stream, with a time delay not exceeding 10 ms. As such, the time delay between the master and slave in this study is deemed negligible. In conclusion, passivity provides sufficient conditions to ensure the stability of the teleoperated robotic control system.

## 4. Results

The experimental results are described in this section, including the experimental setup, the speed mode experiment, the position mode experiment, the corresponding mode switching, and the *Z*-axis force feedback effect.

### 4.1. Experimental Setup

**Figure 11:**
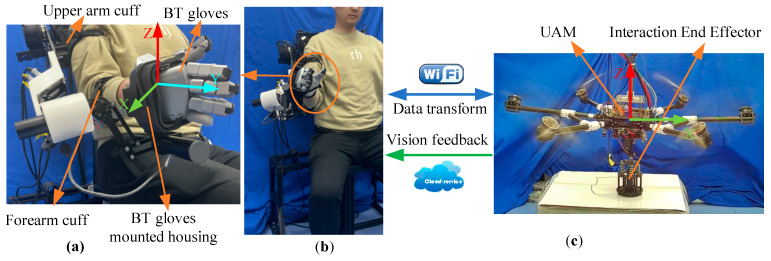
Experimental setup for UAM teleoperated by upper exoskeleton. (**a**) operator wearing exoskeleton; (**b**) master system; (**c**) slave UAM with 3D end-effector.

As illustrated in Figure 11, the operator interfaces with the exoskeleton master robot through an upper arm cuff, a forearm cuff, and a data glove mounting bracket, while the UAM relays visual feedback via a wide-area network server, enabling operation beyond the line of sight. Data communication between the exoskeleton and the unmanned aerial vehicle is facilitated via the TCP/IP protocol over a local area network. The UAM and the end-effector of the exoskeleton are specified within a modified Cartesian coordinate framework.

The experimental protocol proceeds as follows:

(a) Preparation: The UAM operator activates an automated takeoff sequence, directing the UAM to ascend to the designated operational altitude. Upon attaining this altitude, a servo mechanism deploys the 3D end-effector in a perpendicular orientation relative to the operational surface.
(b) Teleoperation: The system subsequently transitions to teleoperation control mode, initiating in the reset state. Thereafter, the operator configures the operational mode using the data glove to switch mode in accordance with the requirements to execute interactive control writing tasks. Upon completion of the teleoperation writing task, the operation mode reverts to the reset mode.
(c) Completion: The UAM operator activates the recovery sequence, retracting the 3D end-effector via the servo system and guiding the UAM to land.

### 4.2. Free Rate Mode Experiment

In the first experiment, the free rate mode was applied for validation of UAM free rate mode results. The control parameters were set as *λ_vt_* = 0.1, *λ_v__θ_* = 0.05, *r_vt_* = 15 mm, *r_v__θ_* = 10°, v_max_ = 0.05 m/s. The selection of these parameters is designed to capture the operator’s real intentions, mitigate the impact of end jitter, and ensure that the slave UAM operates within a reasonable velocity range while maintaining approximately the same tracking error at the slave 3D end-effector. Although the algorithm proposed in this study can be applied to 6 DOF teleoperation mapping for the UAM, given the high sensitivity of the UAM stabilizer attitude control, control parameters were made to minimize the impact of primary control parameters on the stabilizer’s attitude. This enabled the stabilizer to autonomously maintain a posture perpendicular to the horizontal plane. Consequently, the attitude was minimally affected by teleoperation control signals and was not prominently demonstrated in the experiments.

**Figure 12:**
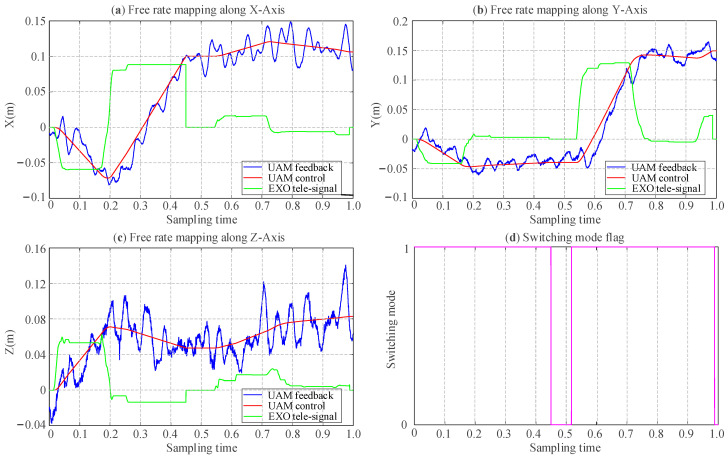
Results of the free rate mode experiment: trajectory tracking performance of master–slave. (**a**) Trajectory tracking alone *X*-axis; (**b**) trajectory tracking alone *Y*-axis; (**c**) trajectory tracking alone *Z*-axis; (**d**) switching mode flag via BT glove.

Figure 12 illustrates the tracking error in free rate mode. Figure 12a–c depicts the master control commands and the UAM position feedback along the *X*, *Y*, and *Z* axes, respectively. The green line represents the master-end velocity commands, the red line indicates the UAM position control commands derived from the integration of velocity commands, and the blue curve corresponds to the UAM real-time position feedback. The master control command (green line) with the area enclosed by the *x*-axis corresponds to velocity, which is reflected in the slope of the red line depicts the UAM control response. However, as the *Z*-axis corresponds to the direction of physical interaction and gravitational influence, the impedance control parameters for the *Z*-axis are distinct, leading to noticeable oscillatory behavior during motion along the *Z*-axis. The impedance controller parameters corresponding to the six degrees of freedom for the UAM are configured as follows: *m_c_* = [4.5, 4.5, 2, 0.085, 0.085, 0.123], *b_c_* = [6, 6, 6, 1.3, 1.3, 1.3], *k_c_* = [8, 8, 12, 1.8, 1.8, 2.0] [41]. It is evident that the UAM achieves effective tracking of the position control commands in the X-Y plane. However, due to differing controller parameter settings for the *Z*-axis, noticeable oscillations are observed in the *Z*-axis motion. Figure 12d illustrates the mode-switching flag generated by the Bluetooth data glove, indicating a reset mode transition at approximately 0.5 sampling time. Consequently, the master-end motion exerts no influence on the UAM motion control, thereby confirming the practical efficacy of the mapping and mode-switching under the free-rate mode.

### 4.3. Position and Feedback Mode Experiment

**Figure 13:**
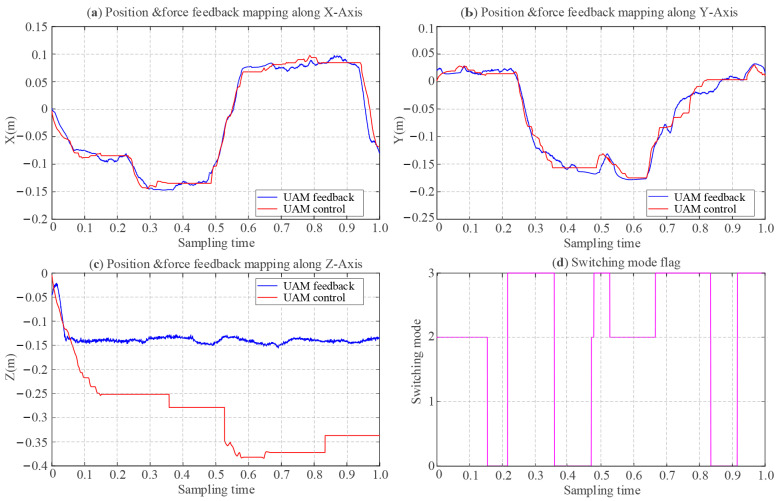
Results of the position and force feedback mode experiment: trajectory tracking performance of master-slave. (**a**) Trajectory tracking alone *X*-axis; (**b**) trajectory tracking alone *Y*-axis; (**c**) trajectory tracking alone *Z*-axis; (**d**) switching mode flag via BT glove.

In the position and feedback mode experiment, the control parameters were set as *λ_pt_* = 1.0, *λ_p__θ_* = 0.2, *r_pt_* = 15 mm, *r_p__θ_* = 10°. Furthermore, due to the tethered configuration of the drone, its operational radius was limited to 0.3 m, with teleoperation position commands that exceed this limit being considered invalid and subsequently limited within a maximum range. As depicted in Figure 13c,d, the switching flag for position mode is designated as 2, while that for force feedback mode is set to 3, respectively. In the experiment, the UAM was initially operated in position control mode to gradually descend until the 3D end-effector reached a contact height of approximately −0.15 m with the whiteboard, at which point the system transitioned to force feedback mode. Notably, upon entering force feedback mode and contact with the whiteboard, the UAM *Z*-axis altitude becomes unresponsive to teleoperation commands, autonomously maintaining the contact height until switching back to position mode, while *X-* and *Y*-axis commands remain effective. Figure 13 shows that position control mode achieves superior tracking accuracy compared to velocity control mode due to the lower motion speed.

**Figure 14:**
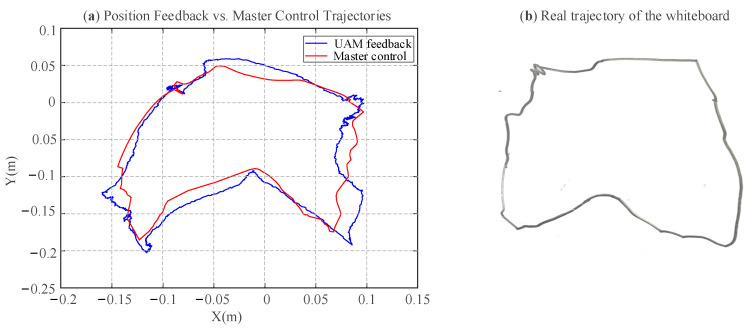
Two-dimensional motion trajectory in the *X*-*Y* plane. (**a**) trajectory of master signal and UAM feedback trajectory; (**b**) real air writing trajectory of the whiteboard pen in the 3D end-effector.

Figure 14a shows the XY-plane projection of the master control and UAM position feedback trajectories, while Figure 14b depicts the real trajectory of 3D end-effector pen on the whiteboard. These trajectories exhibit broadly similar contour shapes, though discrepancies persist in finer details. The position tracking RMSE for position tracking along the *X* and *Y* axes is computed as 7.7% and 5.2%, respectively. The discrepancies observed are primarily attributed to the limited control precision of the UAM, localization inaccuracies stemming from the T265, and frictional effects at the contact interface further compromising trajectory fidelity. The experimental process and results can be visualized in the supplemental video (Video S1 in Supplementary Materials).

**Figure 15:**
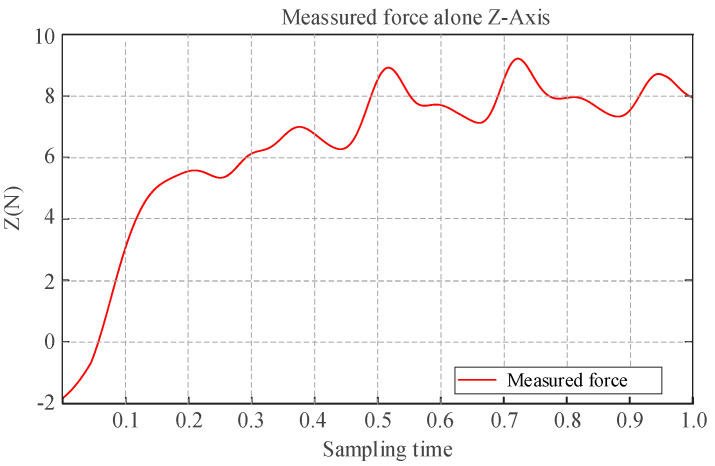
Z-axis contact force measurement curve.

Figure 15 illustrates the force feedback provided during the aerial writing task. The data smoothed via a Gaussian filter with a window size of 100 exhibit frequent and alternating variations in tactile force along the *Z*-axis, indicating dynamic fluctuations in contact interaction. This behavior stems from the application of impedance control along the *Z*-axis, as depicted in Figure 10, which converts the contact force of the UAM along the *Z*-axis into position errors. Additionally, contact force feedback is seamlessly incorporated into the end-effector of the exoskeleton, enabling perception of physical interaction from the slave UAM.

## 5. Discussion

Compared to traditional teleoperation systems based on serial or parallel master robots, this study employs a 7 DOF wearable upper-limb exoskeleton, which aligns naturally with the operator joint angles, offering intuitive control logic and a significantly expanded operational workspace of the master device. Additionally, in contrast to conventional button-based mode switching, this work utilizes Bluetooth data gloves as mode-switching sensors, providing a broader, clearer, and intuitive interaction framework according to the operational task that effectively minimizes the risk of erroneous operations. Combined with the hybrid mapping framework we proposed, the operator can leverage velocity mapping to rapidly approach the target, subsequently transitioning seamlessly to either a heterogeneous position mode or a force feedback mode to perform interactive tasks with enhanced precision control. By wearing the exoskeleton arm and configuring appropriate mapping parameters, operators achieved velocity mapping and interactive aerial writing modes. Experimental findings not only verify the feasibility and stability of the entire system but also demonstrate that the mode-switching algorithm facilitates efficient teleoperation processes. Furthermore, the algorithm proposed herein is not only applicable to UAM teleoperation but, with appropriate parameter adjustments, is equally suitable for mobile manipulator platform.

To quantitatively evaluate the system’s usability, focusing on the intuitiveness and operator comfort, a System Usability Scale (SUS) survey, as is shown in Appendix A, was conducted. The results based on responses from ten participants (nine males and one female) provide a comprehensive evaluation of the exoskeleton for teleoperating the UAM. The mean SUS score was 86.3 (SD = 8.2), surpassing the industry benchmark of 68, indicating above-average usability. Analysis revealed strengths in specific usability dimensions: the statement “The wearable exoskeleton offers enhanced comfort” received a mean score of 4.3, suggesting high user satisfaction with physical ergonomics. Similarly, “The teleoperation mode switching is intuitive and user-friendly” scored 4.8, reflecting intuitive mode transitions, and “The data gloves did not exhibit mode-switching errors during operation” averaged 5.0, confirming reliable performance of mode switching interface. Additionally, “The teleoperation process is free from dizziness or discomfort” scored 4.8, indicating minimal adverse physical effects. However, the statement “The teleoperation process does not exhibit noticeable jitter and unexpected movements” received a lower mean score of 3.9, highlighting a critical area for improvement in control stability. Finally, “The UAM’s trajectory aligns consistently with the master control trajectory” scored 3.1, suggesting generally accurate trajectory tracking but with potential for refinement. These findings validate the system’s intuitive design and comfort while identifying jitter and minor trajectory inconsistencies as priorities for future optimization.

Due to the limited control precision of the UAM, which is significantly lower than the millimeter-level accuracy achievable by robotic manipulators, and the visual localization based on the T265 sensor, constrained to centimeter-level accuracy, experimental trajectories exhibit deviations from actual trajectories, as illustrated in Figure 14. Nevertheless, the aerial writing experiments have comprehensively validated the efficacy of the hybrid mapping algorithm.

## 6. Conclusions

This paper describes a generic haptic teleoperation control architecture for a UAM contact task. Firstly, this paper employs an upper arm exoskeleton as the user-friendly master robot, offering superior human adaptability and a broader workspace compared to conventional desktop level haptic devices. Then, to address the requirements of workspace and accuracy in UAM contact tasks, this work introduces a continuous rate and position control mode that innovatively employs a bluetooth data glove as the command-switching device. The proposed control architecture adeptly addresses challenges in teleoperation, including misoperations arising from non-intuitive mode switching, oscillations triggered by mode switching, and limitations in the workspace of master devices, thereby significantly enhancing system stability and operational efficacy. Finally, a comprehensive experimental platform was established, and an aerial writing experiment was conducted. The results showed that the trajectory tracking error of the areal writing experiment achieved a position tracking RMSE of 7.7% and 5.2% in the *X*,*Y*-axis, respectively. Additionally, the smooth UAM motion observed during the experiments, with no abrupt feedback force variations, further highlights the excellent stability of the control framework, which has potential applications in autonomous aerial vehicles and human–machine interaction. The SUS survey effectively validates the intuitive and comfort design of the teleoperation master system.

In the future, we plan to implement the teleoperation system in a six-dimensional workspace and on a broader spectrum of robotic platforms to further evaluate its universality.

## Figures and Tables

**Table 1:** Modified D-H parameters of the right exoskeleton.

Link *i*	*θ*	*d*	*a*	*α*	Offset	Range (°)
1	*θ*_1_	−*l_a_*	*l_b_*	−pi/2	0	−90~0
2	*θ*_2_	0	0	pi/2	pi/2	−36~79
3	*θ*_3_	0	0	−pi/2	pi/2	−90~0
4	*θ*_4_	0	*l_c_*	0	0	−142~0
5	*θ*_5_	0	*l_d_*	0	−pi/2	−30~40
6	*θ*_6_	0	0	pi/2	pi/2	−55~40
7	*θ*_7_	0	0	pi/2	0	−45~60

## Data Availability

The original contributions presented in this study are included in the article/Supplementary Material. Further inquiries can be directed to the corresponding author.

## Supplementary Materials

The following supporting information can be downloaded at https://www.mdpi.com/article/10.3390/s25185844/s1, Video S1: Video of the verification experiment for bilateral teleoperation of aerial manipulators based on the hybrid mapping framework.

## Appendix A

### Exoskeleton Teleoperation User Survey Questionnaire

A System Usability Scale (SUS) survey was conducted to systematically evaluate the user experience of the wearable upper arm exoskeleton for teleoperating the Unmanned Aerial Manipulator (UAM). We recruited ten participants (including nine males and one female) to perform UAM teleoperation using the exoskeleton and data gloves as mater robot. Subsequently, the following feedback questionnaire incorporating evaluations of the experimental results and comfort level after experiment are completed.

**Table A1:** The system usability scale questionnaire of the teleoperation system. For each of the following statements, mark one box that best describes your reaction to the teleoperation experiment today.

Question Options	Strongly Disagree				Strongly Agree
The wearable exoskeleton robotic arm offers enhanced comfort	□	□	□	□	□
The teleoperation mode switching is intuitive and user-friendly	□	□	□	□	□
The data gloves do not exhibit mode-switching errors during operation	□	□	□	□	□
The teleoperation process is free from dizziness or discomfort	□	□	□	□	□
The teleoperation does not process exhibit noticeable jitters and unexpected movements	□	□	□	□	□
The UAM’s trajectory aligns consistently with the master control trajectory	□	□	□	□	□
